# *Bacillus cereus* EC9 protects tomato against Fusarium wilt through JA/ET-activated immunity

**DOI:** 10.3389/fpls.2022.1090947

**Published:** 2022-12-15

**Authors:** Sercan Pazarlar, Kenneth Madriz-Ordeñana, Hans Thordal-Christensen

**Affiliations:** ^1^ Department of Plant Protection, Faculty of Agriculture, Ege University, Izmir, Turkey; ^2^ Department of Plant and Environmental Sciences, Section for Plant and Soil Science, University of Copenhagen, Copenhagen, Denmark

**Keywords:** *Fusarium oxysporum* f. sp. *lycopersici*, rhizobacteria, biological control, defense priming, induced resistance, phytohormones

## Abstract

The mechanisms of action and the limitations of effectiveness of natural biocontrol agents should be determined in order to convert them into end products that can be used in practice. Rhizosphere *Bacillus* spp. protect plants from various pathogens by displaying several modes of action. However, the ability of *Bacillus* spp. to control plant diseases depends on the interaction between the bacteria, host, and pathogen, and the environmental conditions. We found that soil drenching of tomato plants with the non-antifungal *Bacillus cereus* strain EC9 (EC9) enhances plant defense against *Fusarium oxysporum* f. sp. *lycopersici* (*Fol*). To study the involvement of plant defense-related phytohormones in the regulation of EC9-activated protection against *Fol*, we conducted plant bioassays in tomato genotypes impaired in salicylic acid (SA) accumulation, jasmonic acid (JA) biosynthesis, and ethylene (ET) production, and analyzed the transcript levels of pathways-related marker genes. Our results indicate that JA/ET-dependent signaling is required for EC9-mediated protection against *Fol* in tomato. We provide evidence that EC9 primes tomato plants for enhanced expression of *proteinase inhibitor I* (*PI-I*) and *ethylene receptor4* (*ETR4*). Moreover, we demonstrated that EC9 induces callose deposition in tomato roots. Understanding the involvement of defense-related phytohormones in EC9-mediated defense against Fusarium wilt has increased our knowledge of interactions between non-antifungal plant defense-inducing rhizobacteria and plants.

## 1 Introduction

Tomato (*Solanum lycopersicum* L.), as a highly important crop for direct consumption and raw material for various products, has been cultivated extensively in many parts of the world for decades (Costa and Heuvelink, 2018). Fusarium wilt of tomato caused by the soil-borne ascomycete fungus, *Fusarium oxysporum* Schlecht f. sp. *lycopersici* (*Fol*), is one of the most destructive diseases of tomato (McGovern, 2015; Nirmaladevi et al., 2016). *Fol* is a hemibiotroph that penetrates through wounds at the root tip and lateral root formation zones. It colonizes the apoplast of the root cortex, and invades the xylem vessels, leading to wilting, yellowing, vascular discoloration, growth distortion, and eventually to the death of the plant (Di Pietro et al., 2003; Michielse and Rep, 2009; de Lamo and Takken, 2020). Chemical control of Fusarium wilt of tomato is not effective due to poor delivery of conventional fungicides to the xylem vessels, not to mention difficulties with soil treatments. Additionally, *Fol* chlamydospores are highly infectious and can survive on plant debris for long time, making disease control very challenging (De Cal et al., 1997; Hou et al., 2020). Meanwhile, one way of controlling *Fol* is through resistance breeding. Three Secreted In Xylem (SIX) effectors (Six1, Six3, and Six4), are required for virulence of *Fol* (Rep et al., 2005; Houterman et al., 2008; Houterman et al., 2009). These effectors, also referred to as Avr3, Avr2, and Avr1, are recognized by the intracellular resistance (R) proteins Immunity-3 (I-3), I-2, and I, respectively, in tomato (de Sain and Rep, 2015). Accordingly, *Fol* is differentiated into races 1, 2, and 3 based on its ability to overcome the corresponding *R* genes (Biju et al., 2017). However, when these *R* genes are combined in cultivars, novel *Fol* strains overcoming these may emerge (Takken and Rep, 2010).

In recent years, biological control of Fusarium wilt of tomato has attracted considerable attention (La Torre et al., 2016; Sallam et al., 2019; de Lamo and Takken, 2020; Doan et al., 2020; Vinchira-Villarraga et al., 2021). Several species of the bacterial genus *Bacillus* have proved to be promising biocontrol agents, since they protect through several modes of action, such as competition, parasitism, antibiosis, and induced resistance (Akram et al., 2016; Elanchezhiyan et al., 2018; Bhattacharya et al., 2019; Medeiros and Bettiol, 2021; Zhang et al., 2021). For the latter mechanism, some *Bacillus* species can activate immediate defense responses, predisposing the plant to react faster and stronger to subsequent pathogen attack, a mechanism known as defense priming (Tonelli and Fabra, 2014; Wang et al., 2014; Mauch-Mani et al., 2017).

Plants sense an attacking pathogen through recognition of microbe-associated molecular patterns (MAMPs), leading to pattern-triggered immunity (PTI). As a counterattack, effector proteins secreted by the pathogen hamper PTI to re-establish susceptibility. In turn, the effectors can be recognized by specific plant R receptors resulting in effector-triggered immunity (ETI), which is associated with local programmed cell death (Ngou et al., 2022). In addition, systemic defense pathways can be induced in the plant. Two main such interacting defense pathways have been described: “systemic acquired resistance” (SAR) and “induced systemic resistance” (ISR) (Conrath, 2006; Pieterse et al., 2014). Salicylic acid (SA), synthesized *via* isochorismate synthase or phenylalanine ammonium lyase, is a key phytohormone regulating the SAR reactions initiated by pathogens and various natural and synthetic elicitors (Ngou et al., 2022). On the other hand, jasmonic acid (JA) and ethylene (ET) mediate ISR, which is typically activated by nonpathogenic (and beneficial) rhizobacteria such as *Bacillus* spp. and *Pseudomonas* spp. (Bakker et al., 2007; Blake et al., 2021). NONEXPRESSOR OF PATHOGENESIS-RELATED GENES 1 (NPR1) regulates SAR by recruiting TGACG-Binding (TGA) transcription factors to, for instance, activate *PR* genes that encode antimicrobial proteins and other genes that act as key elements in the crosstalk between SA and JA/ET-mediated reactions (Backer et al., 2019).

*Bacillus* spp. activate ISR in many plant species such as *Arabidopsis thaliana* (Nie et al., 2017 *)*, tobacco (Huang et al., 2012), tomato (Yoshida et al., 2019), maize (Xie et al., 2019), and rose (Chen et al., 2020). *Bacillus*-mediated defense responses depend not only on the transduction of JA/ET signaling, but also on SA-dependent signaling (Niu et al., 2011; Xie et al., 2021; Chaparro-Encinas et al., 2022). In addition, MAMPs and several metabolites produced by *Bacillus* spp., including well-known antimicrobial compounds such as surfactin and fengycins, have been shown to boost plant defense by activating ISR (Ongena et al., 2007; Jiang et al., 2016). Furthermore, volatile organic compounds from *Bacillus* spp. can also activate ISR (Rudrappa et al., 2010; Yi et al., 2016). Overall, the ability of *Bacillus* spp. to control plant diseases depends on the interaction between the bacteria, host, and pathogen, and the environmental conditions.

Recently, we reported that the antifungal *B. siamensis* strain DD6 and the non-antifungal *B. cereus* strain EC9 effectively suppressed root and stem rotting disease caused by *F. oxysporum* in the ornamental plant Kalanchoe (*Kalanchoe blosssfeldiana*) (Madriz-Ordeñana et al., 2022). Furthermore, expression analysis of the defense-related genes *PR1* and *LOX2* revealed activation of a primed state in Kalanchoe when the roots were colonized by EC9. These results prompted us to study the involvement of defense-related phytohormones in EC9-activated protection in the *Fol*/tomato pathosystem. In this study, we conducted plant bioassays in tomato genotypes impaired in SA accumulation, JA biosynthesis, and ET production, and analyzed the expression levels of pathway-related marker genes. We provide evidence indicating that JA/ET signaling is necessary for the biocontrol of Fusarium wilt of tomato activated by EC9. Furthermore, we show that EC9 primes the expression of JA/ET-related marker genes and induces callose deposition in tomato roots.

## 2 Materials and methods

### 2.1 Plant materials and growth conditions

The *NahG* transgenic tomato line (cv. Moneymaker), impaired in SA accumulation by expressing the *salicylate hydroxylase* gene (Brading et al., 2000), was kindly provided by Prof. Jonathan Jones (The Sainsbury Laboratory, Norwich, UK). The JA-deficient *spr2* (*suppressor of prosystemin-mediated responses2*) mutant (Howe and Ryan, 1999) and the corresponding wild-type cv. Castlemart were kindly provided by Dr. María Fernanda López Climent (Jaume I University, Castelló de la Plana, Spain). Transgenic *ACD* tomato, constitutively overexpressing (OE) the bacterial ACC deaminase gene resulting in compromised ET production (Klee et al., 1991), and the corresponding wild-type cv. UC82B were obtained from Dr. Birgit Jensen (University of Copenhagen, Denmark). The cv. Moneymaker was routinely used to analyze the expression pattern of marker genes for relevant phytohormones and to monitor callose deposition in roots. In all experiments, seeds were surface sterilized with 2.5% NaClO (v/v) and 0.05% Tween 20 for 15 min and 70% EtOH (v/v) for 1 min, followed by at least 3 times washing in distilled water. Sterilized seeds were planted in plastic pots containing peat moss substrate (Klasmann TS1, Germany). Plants were grown at 23°C on a 16/8 h day/night cycle with supplemental lightning of 160 μmol m^-2^ s^-1^ in the greenhouse.

### 2.2 Preparation of bacterial suspensions and fungal inoculum

*B. cereus* EC9 and *B. siamensis* DD6 strains were previously isolated from Kalanchoe-associated materials (Madriz-Ordeñana et al., 2022). The bacterial strains were grown on Luria-Bertani (LB) (Sigma-Aldrich, Germany) agar plates at 28°C for 24 h and a single colony was used to inoculate 50 ml of LB. The cultures were incubated at 28°C with a constant shaking at 125 rpm for 24 h. The cells were collected by centrifugation at 6000x g for 10 minutes and resuspended in 10 mM MgCl_2_.

*Fol* 4287 (race 2) was kindly provided by Prof. Martijn Rep (University of Amsterdam, The Netherlands). The fungus was cultured on potato dextrose agar (PDA) (Scharlau Chemie, Spain) at 25°C for 7 days. Mycelial plugs (9 mm ø) were transferred to 100 ml of minimal medium (1% KNO_3_, 3% sucrose and 0.17% Yeast Nitrogen Base without amino acids and ammonia) and incubated at 25°C with shaking at 150 rpm for 5 days. The spore suspension was filtered through four layers of sterile cheesecloth, centrifuged at 5000x g for 10 min and rinsed with sterile distilled water. The final concentration was adjusted to 1x10^7^ spores ml^-1^ (Di et al., 2017).

### 2.3 Antagonistic assay of *Bacillus* spp. against *Fol*

The agar disk diffusion method described by Shehata et al. (2016) was applied to test the *in vitro* antagonistic efficacy of DD6 and EC9. Briefly, the spore suspension of *Fol* prepared as described above was adjusted to a final concentration of 1x10^5^ spores ml^-1^ in freshly prepared and cooled PDA (~50°C) and poured into each sterile Petri dish. One hundred microliters of each bacterial LB culture at OD_600_ 0.25, 0.5, 0.75, and 1 were pipetted into 15 mm ø wells created in the PDA plates using a sterilized glass tube. Sterile LB medium was used as control. The antifungal activity of strains was determined by measuring the distance between opposed edges of the inhibition zone of each well (excluding the diameter of wells) after 5 days of incubation.

### 2.4 Protection efficacy *in planta* of strains DD6 and EC9 against *Fol*

Ten-days-old tomato plants were treated with 5 ml cell suspension of each strain at OD_600_ of 0.3 or 10 mM MgCl_2_ (mock) by soil drenching. After one week, the plants were up-rooted and either inoculated with *Fol* spore suspension (1x10^7^ spores ml^-1^) or treated with water (for mock inoculation) by the root dipping method (Constantin et al., 2020). The inoculated plants were replanted on the same peat substrate. Disease severity (DS) was scored 3 weeks after inoculation according to the following scale (Constantin et al., 2019): DS0 = no symptoms; DS1 = brown vessel at the crown level; DS2 = one or two brown vessels at the cotyledon level and no outer symptoms; DS3 = three or more brown vessels with external wilting and growth distortion symptoms, DS4 = all vessels brown and severe wilting/stunning symptoms, DS5 = plant is dead.

For *Fol* recovery assay, approximately 3 mm thick tomato stems were sectioned using a microtome blade at the levels of the crown, cotyledon, 2nd and 4th nodes, and surface sterilized with 5% NaClO (v/v) for 15 min and 70% EtOH (v/v) for 1 min, then rinsed with sterile distilled water for 3 min at least three times. After air drying, the stem sections were placed on PDA medium under sterile conditions. The plates were incubated for 5 days at 25°C and the percentage of *Fol* colonization was expressed based on the outgrowth of fungal mycelium from stem sections (Di et al., 2017).

### 2.5 Quantification the biomass of *Fol* in tomato plants

To determine the relative amount of *Fol* biomass in the vascular tissue, sections of stems taken between the crown and the cotyledon of individual plants were used for genomic DNA extraction using the Plant DNeasy kit (Qiagen GmbH, Germany). *Fol* biomass estimation was performed by qPCR on the extracted DNA using tomato and fungal specific primers (**Supplementary Table S1**) in the LightCycler96 System (Roche Diagnostics GmbH, Germany). PCR reactions were performed using the HOT FIREPol EvaGreen qPCR Mix Plus following the conditions recommended by the manufacturer (Solis BioDyne, Tartu, Estonia). Fungal biomass was estimated by calculating the ratio of fungal DNA to tomato DNA using serial dilutions from 0.002 to 20 ng of pure genomic DNA of each organism. Standard curves were fit by linear regression, and the amount of DNA was estimated by tracing the Ct-values against the corresponding known amounts of DNA.

### 2.6 Analysis of defense gene expression

Total RNA was extracted from the segment of the tomato stem between the cotyledons and the crown at 3, 7, 14 dpi using TRIzol reagent (TRI Reagent, R2050-1-200, Zymo Research) following the instructions provided by the manufacturer. Two stems randomly selected from different plants were pooled for RNA extraction. First-strand cDNA was synthesized from 1 µg RNA using a RevertAid First Strand cDNA Synthesis Kit (K1622, ThermoFisher Scientific, Walthamm MA, USA) with oligo(dT) primers, and the qRT-PCR assay was performed using EvaGreen 2× qPCR MasterMix (MasterMix-R, abm, Canada) in a PikoReal 96 real-time PCR system (Thermo Scientific, Burlington, Canada). Twenty microliter reaction mixtures consisted of 10 µl EvaGreen 2X qPCR MasterMix, volume of cDNA corresponding to 250 ng in final concentration, and 0.3 µM of reverse and forward primers. The conditions for PCR cycling were as follows: 95°C for 15 min and 40 cycles of 95°C for 15 s, 60°C for 20 s, and 72°C for 30 s. Relative transcript levels were calculated as described by Pfaffl (2001) and the values were normalized to the internal reference gene *α-tubulin*. The primer sequences for qRT-PCR used are listed in **Supplementary Table S1**.

### 2.7 Visualization of callose deposition in the roots

Sterilized tomato seeds were grown in vertically placed square Petri dishes, containing ½ x Murashige and Skoog medium (containing 1% sucrose) supplemented with 0.8% agar (pH: 5.7), in a growth chamber at 20°C and a 16/8 h day/night cycle (200 μmol m^-2^ s^-1^). Seven-days-old tomato seedlings were lifted from the plates and the roots were dip treated with EC9 suspension or mock for 1 min. and returned to growth media. Twenty-four hours after treatment, the roots were dip-inoculated with *Fol* conidia suspension or sterilized water for 1 min. and replated. Roots were collected 24 h after inoculation and fixed in acetic acid:ethanol (1:3 v/v) for 4 h, followed by staining with aniline blue (0.1% w/v) in 150 mM K_2_HPO_4_ overnight in the dark (Schenk and Schikora, 2015). The roots were then mounted on a microscope slide and examined using a Leica DM 5000B fluorescence microscope using a DAPI filter. The number of callose spots was determined in the root segment between 1 and 2 cm from the primary tip.

### 2.8 Statistical analysis

All statistical analyzes were performed with Graph Pad Prism v.9 (San Diego, CA, USA) and SPSS v.25 (IBM, Armonk, NY, USA) software. Normality and homogeneity of variance were tested by the Shapiro-Wilk test. The effects of treatments on disease incidence, fresh weights, fungal recovery, *in vitro* antifungal effect, relative quantification of *Fol*, callose deposition were analyzed with the Student’s *t*-test. If the data were not distributed normally, we applied the Mann-Whitney *U-*test. Comparison of the effects of the treatments on the gene expression profile of tomato plants was performed using one-way analysis of variance (ANOVA) and Duncan’s *post hoc* multiple comparisons test. The data presented here were confirmed by at least two independent experiments.

## 3 Results

### 3.1 *B*. *cereus* EC9 protects tomato plants from *Fol* infection but does not show antifungal activity *in vitro*

**Figure 1:**
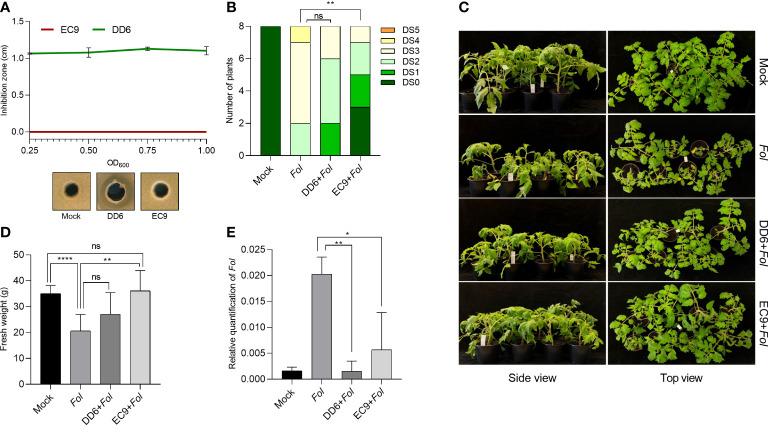
Treatment with *B cereus* EC9 enhances resistance to *Fusarium* wilt in tomato. **(A)** Inhibition zones caused by *Bacillus* spp. against *Fol* on PDA medium. The antifungal activity was determined after 5 days of incubation (n=5). **(B)** Disease severity score, **(C)** representative pictures of disease symptoms, and **(D)** fresh weight of tomato plants (cv. Moneymaker) at 21 days post-inoculation (dpi) (n=8). Ten days old tomato seedlings were treated with DD6, EC9 or 10 mM MgCl_2_ (mock) by soil drenching and inoculated with *Fol* 1 week after treatment. **(E)** Estimation *in planta* (n=4) of *Fol* biomass in tomato stems. Quantification of *Fol* was carried out in plants at 21 dpi by calculating the ratio of fungal DNA to tomato DNA by qPCR. ns non-significant, *P< 0.05, **P < 0.01, ****P < 0.0001. The experiments were repeated at least two times with similar results. Bars represent the means of the indicated number of biological replicates ± standard error.

We wanted to establish whether the antifungal and non-antifungal activity of DD6 and EC9, respectively, found in the *F. oxysporum*/Kalanchoe interaction (Madriz-Ordeñana et al., 2022), also applies to Fusarium wilt on tomato. Thus, we evaluated the fungal growth inhibition activity *in vitro*, as well as the development of disease symptoms in bacteria-treated plants 3 weeks after *Fol* inoculation. While DD6 strongly inhibited the mycelial growth of *Fol* at all the bacterial densities tested, the lack of inhibition of *Fol* growth by EC9 was not distinct from the control (**Figure 1A**). The evaluation *in planta* showed that for EC9, the development of symptoms, including vessel browning, stunting, growth distortion, and wilting, was greatly reduced in comparison with the mock-treated plants (**Figures 1B, C**). Similarly, treatment with EC9 resulted in higher fresh weight (**Figure 1D**) and a significantly lower amount of *Fol* biomass in tomato stems (**Figure 1E**). In contrast, treatment with the antifungal strain DD6 did not significantly reduce symptom development nor increased the fresh weight of the infected plants (**Figures 1B–D**). However, a lower level of fungal biomass was detected in DD6-treated plants in comparison to mock inoculated plants (**Figure 1E**).

### 3.2 *B. cereus* EC9-mediated protection is independent of SA signaling

**Figure 2:**
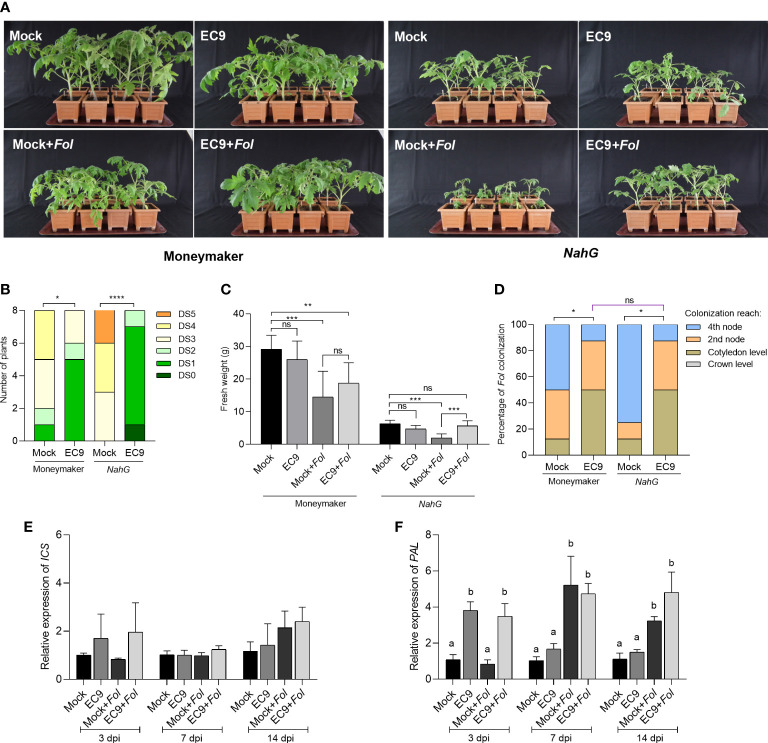
The SA signaling pathway is not involved in EC9-enhanced defense in tomato. **(A)** Disease symptoms, **(B)** disease severity score and **(C)** fresh weight of transgenic *NahG* plants and the corresponding wild type cv. Moneymaker at 21 days post-inoculation (dpi) (n=8). Ten-days old tomato seedlings were treated with EC9 or 10 mM MgCl_2_ (mock) by soil drenching and inoculated with *Fol* 1 week after treatment. **(D)** Percentage of *Fol* infected stem sections at 21 dpi (n=8). ns non-significant, *P < 0.05, **P < 0.01, ***P < 0.001, ****P<0.0001. The accumulation of transcripts of **(E)** *isochorismate synthase* (*ICS*) and **(F)** *phenylalanine ammonia-lyase* (*PAL*) in tomato plants (cv. Moneymaker) treated with EC9 or mock and inoculated with *Fol* was revealed by qRT-PCR (n=3). For gene expression analysis, stem pieces from the region between cotyledons and crown were harvested. The values were normalized to the internal reference transcript *α-tubulin.* Bars represent the means of the indicated number of biological replicates ± standard error. The experiments were repeated at least two times with similar results. Different letters indicate statistically significant differences between treatments.

We further intended to determine the involvement of plant defense hormone signaling pathways in EC9-mediated protection against *F. oxysporum* in tomato. Thus, we first investigated whether EC9-mediated protection involves SA-signaling. Here we made use of an SA-compromised *NahG* transgenic tomato line (Brading et al., 2000). As expected from Di et al. (2017), untreated *NahG* plants showed increased susceptibility to *Fol* compared to the wild-type cv. Moneymaker (**Figures 2A–C**). At the same time, disease severity in cv. Moneymaker was significantly reduced by treatment with EC9. However, this was also the case in EC9-treated *NahG* plants exhibiting reduced Fusarium wilt and increased fresh weight compared to the mock (**Figures 2A–C**). In addition, the fungal recovery assay revealed that EC9 treatment caused *Fol* to reach the upper parts of the stem only in a reduced number of plants in both cv. Moneymaker and *NahG* line (**Figure 2D**). To gain further insight into the role of SA signaling in EC9-mediated protection, the expression level of the SA biosynthesis-associated genes *ICS* and *PAL* was examined in EC9-treated and untreated cv. Moneymaker plants subsequently challenged with *Fol*. The level of the *ICS* and *PAL* transcripts are commonly used to indicate SA-responses in tomato (Di et al., 2017; Constantin et al., 2019). None of the treatments induced a significant change in the level of the *ICS* transcript at any of the tested time points (**Figure 2E**). For *PAL*, however, the transcript level in plants treated with EC9 alone or in combination with *Fol* moderately increased at 3 dpi. At 7 and 14 dpi, an increased PAL transcript level was observed only in *Fol*-inoculated plants, whether or not treated with EC9 (**Figure 2F**).

### 3.3 JA pathway is required for *B. cereus* EC9-mediated protection against *Fol*

**Figure 3:**
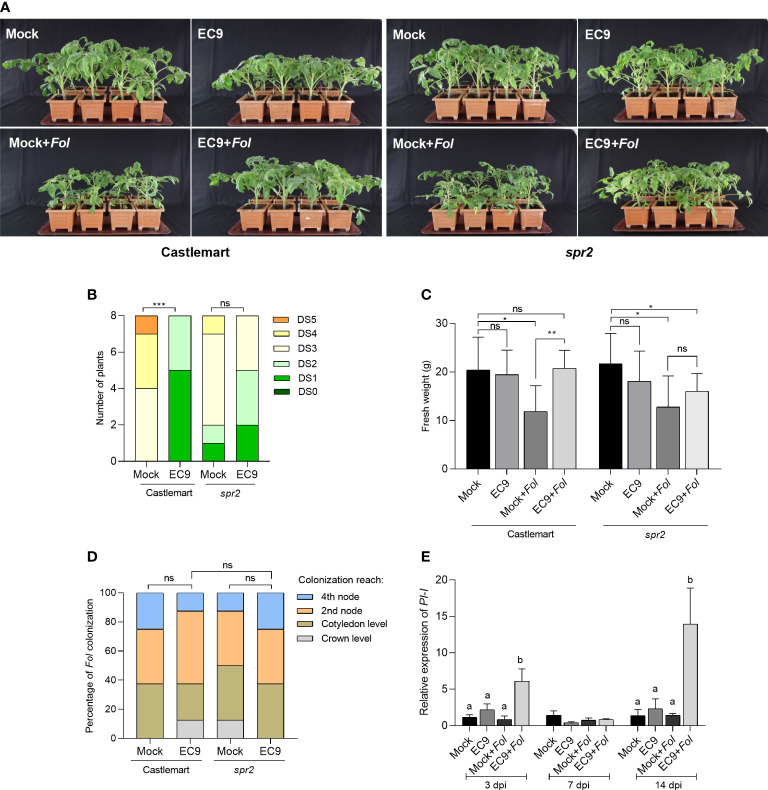
EC9-mediated enhanced defense against *Fol* involves JA signaling pathway. **(A)** Disease symptoms, **(B)** disease severity score, and **(C)** fresh weight of *Spr2* mutant and the corresponding wild type cv. Castlemart at 21 days post-inoculation (dpi) (n=8). Ten-days old tomato seedlings were treated with EC9 or 10 mM MgCl_2_ (mock) by soil drenching and inoculated with *Fol* 1 week after treatment. **(D)** Percentage of *Fol* infected stem sections at 21 dpi (n=8). ns non-significant, *P < 0.05, **P < 0.01, ***P < 0.001,. The accumulation of transcripts of **(E)** *proteinase inhibitor I* (*PI-I*) in tomato plants (cv. Moneymaker) treated with EC9 or mock and inoculated with *Fol* was revealed by qRT-PCR (n=3). For gene expression analysis, stem pieces from the region between cotyledons and crown were harvested. The values were normalized to the internal reference transcript *α-tubulin.* Bars represent the means of the indicated number of biological replicates ± standard error. The experiments were repeated at least two times with similar results. Different letters indicate statistically significant differences between treatments.

Next, we turned to study the involvement of JA in the enhanced protection mediated by EC9 using the JA-deficient tomato line *spr2* (Howe and Ryan, 1999). Inoculation with *Fol* resulted in clear symptoms at 21 dpi in untreated *spr2* and the respective wild-type cultivar Castlemart, suggesting that none of these genotypes exhibited resistance to *Fol* (**Figure 3A**). While most of the wild-type plants showed severe disease symptoms, such as growth distortion and wilting at 21 dpi, disease development was significantly reduced in plants treated with EC9 (**Figures 3A, B**). In addition, **cv.** Castlemart plants treated with EC9 and inoculated with *Fol* showed higher fresh weight than mock-treated, *Fol*-inoculated plants (**Figure 3C**). In contrast, EC9 treatment of *spr2* plants neither changed the level of Fusarium wilt significantly (**Figures 3A, B**) nor affected their fresh weight (**Figure 3C**). In addition, *Fol* colonization of the main stem was not affected by EC9 treatment in cv. Castlemart and *spr2* line (**Figure 3D**). The transcript level of JA signaling marker gene *PI-I* (Di et al., 2017) was monitored in stems of cv. Moneymaker plants where the roots were EC9-treated and mock-treated, 3, 7, and 14 days after *Fol* challenge. EC9 treatment alone did not affect the *PI-I* transcript level at the time points tested. Following *Fol* inoculation, however, EC9-treated plants exhibited a higher transcript level of *PI-I* at 3 and 14 dpi (**Figure 3E**).

### 3.4 *B. cereus* EC9 protects tomato plants against *Fol* in an ET signaling-dependent manner

**Figure 4:**
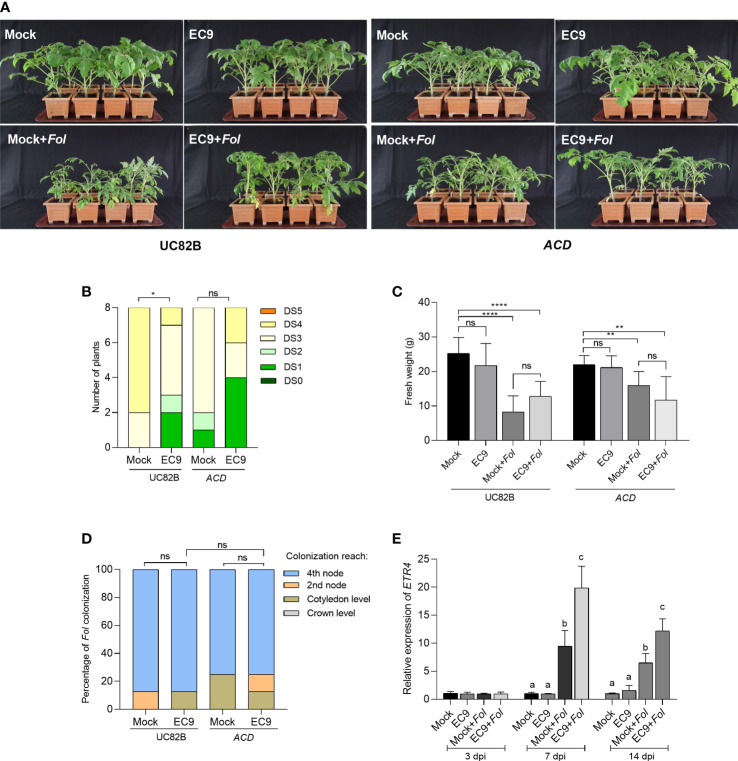
ET is involved in EC9-mediated enhanced defense against *Fol.* **(A)** Disease symptoms, **(B)** disease severity score, and **(C)** fresh weight of *ACD* transgenic line and the corresponding wild type cv. UC82B at 21 days post-inoculation (dpi) (n=8). Ten-days old tomato seedlings were treated with EC9 or 10 mM MgCl_2_ (mock) by soil drenching and inoculated with *Fol* 1 week after treatment. **(D)** Percentage of *Fol* infected stem sections at 21 dpi (n=8). ns non-significant, *P < 0.05, **P < 0.01, ****P < 0.0001. **(E)** The accumulation of transcripts of *ethylene receptor 4* (*ETR4)* in tomato plants (cv. Moneymaker) treated with EC9 or mock and inoculated with *Fol* was revealed by qRT-PCR (n=3). For gene expression analysis, stem pieces from the region between cotyledons and crown were harvested. The values were normalized to the internal reference transcript *α-tubulin.* Bars represent the means of the indicated number of biological replicates ± standard error. The experiments were repeated at least two times with similar results. Different letters indicate statistically significant differences between treatments.

JA signaling is partly linked to ET signaling (Robert-Seilaniantz et al., 2011). Therefore, we wanted to determine whether the ET signaling also is involved in the protection mediated by EC9. This was studied using the ET compromised tomato transgenic line overexpressing *ACD* (Klee et al., 1991). *Fol* inoculation of the *ACD* OE line and the corresponding wild-type cultivar UC82B plants showed clear disease symptoms at 21 dpi (**Figure 4A**). In addition, a significant reduction in disease severity was observed in EC9-treated UC82B plants, confirming that this line is useful for studying EC9 function. In contrast, no significant enhanced protection was observed in the *ACD* OE line (**Figures 4A, B**). The fresh weight and *Fol* colonization of the main stem were not significantly affected by EC9-treatment in cv. UC82B and *ACD* OE line (**Figures 4C, D**). We performed qRT-PCR to measure the transcript level of the ET signaling marker gene *ETR4* (Di et al., 2017) in stems 3, 7, and 14 days after *Fol* challenge of cv. Moneymaker plants, root-treated with EC9 and mock-treated. Treatment with EC9 alone did not trigger any changes in the *ETR4* transcript level at the tested time points, whereas *Fol* inoculation alone resulted in higher *ETR4* transcript accumulation at 7 and 14 dpi. However, when also treated with EC9, *Fol*-inoculated plants showed increased *ETR4* transcript levels at 7 and 14 dpi, in comparison to *Fol* inoculation alone (**Figure 4E**).

### 3.5 *B. cereus* EC9 induces callose deposition in tomato roots

**Figure 5:**
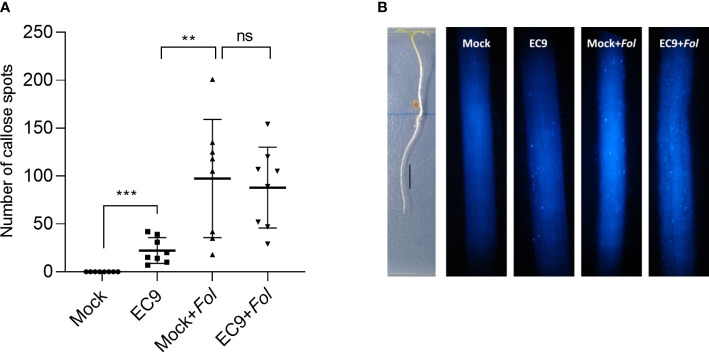
*B cereus* EC9 induces callose deposition in tomato roots. **(A)** Number of the callose spots in roots (cv. Moneymaker) (n=8-10) 24 hours post-inoculation. **(B)** Representative images of tomato roots of 7 days old tomato plants, grown on ½ x MS medium positioned vertically, treated with EC9 or mock and inoculated with *Fol* at 24 hours post-treatment. Presence of callose spots were visualized by aniline blue staining and fluorescence microscopy. ns non-significant, **P < 0.01, ***P < 0.001. Data points correspond to the mean ± standard error. Experiments were repeated two times with similar results.

To analyze whether EC9 activates immune responses in roots, which is the site of *Fol*’s primary attack, we studied the level of callose in this tissue. Callose deposition was determined in the elongation zone of EC9-treated and untreated cv. Moneymaker tomato roots 24 hours after *Fol* inoculation. Treatment with EC9 alone resulted in significantly increased callose deposition. Roots inoculated with *Fol* had an even higher number of callose spots, whether or not pre-treated with EC9 (**Figures 5A, B**).

## 4 Discussion

*Bacillus*-mediated plant disease protection can rely on direct effects of bacteria-derived antimicrobial metabolites on pathogens, widely known as antibiosis, or indirect effects by activating defense in the host plant (Pérez-García et al., 2011; Blake et al., 2021). We have previously identified antifungal (*B. siamensis* DD6) and non-antifungal (*B. cereus* EC9) strains and have shown that both strains provide enhanced protection against Fusarium disease in Kalanchoe (Madriz-Ordeñana et al., 2022). In the present study, we firstly demonstrate that treatment of tomato plants with the antifungal strain DD6 did not significantly reduce *Fol* symptom development despite showing strong antifungal effect *in vitro*. However, we could confirm our previous findings on the role of the non-antifungal strain EC9 as a biocontrol agent (**Figure 1**). To explore whether the mechanisms behind the EC9-mediated protection against Fusarium wilt of tomato involves immunity and whether it involves one or more of the major plant hormone signaling pathways, we conducted plant assays in different phytohormone impaired genotypes and analyzed the expression of related marker genes. Our results suggest that EC9 enhances defense against *Fol* in tomato, possibly by priming of the JA/ET signaling pathways rather than the SA pathway.

The increasing demand for commercial products that are based on biocontrol agents such as *Bacillus* spp. requires that the mechanisms underlying *Bacillus*-mediated protection are properly investigated for various *Bacillus*-plant-pathogen interactions. Successful commercialization of *Bacillus* spp. has so far relied mostly on their production of antimicrobial metabolites capable of directly inhibiting the pathogen. The ability of members of the *B. subtilis*-like group to biosynthesize specialized compounds with antimicrobial activity have made this species attractive for use as biocontrol agents (Caulier et al., 2019; Penha et al., 2020). Thus, treatment of tomato seeds with antifungal *B. velezensis* AP3 (Medeiros and Bettiol, 2021), *B. subtilis* MMS9 (Patel and Saraf, 2017), and *B. amyloliquefaciens* FZB24 (Elanchezhiyan et al., 2018) suppressed Fusarium wilt of the plant. In our study, the antifungal DD6, which contains biosynthetic gene clusters (BGC) associated with well-known antimicrobial secondary compounds, was unable to significantly decrease the development of symptoms of Fusarium wilt disease in tomato (**Figure 1**). This result contradicts our previous findings (Madriz-Ordeñana et al., 2022) showing that DD6 treatment by soil drenching effectively controls *F. oxysporum* in Kalanchoe. This inconsistency for DD6 may be attributed to differences in bacteria-plant species adaptation, root bacterial colonization patterns and environmental conditions. Interestingly, although DD6 treatment did not result in a reduction in Fusarium wilt disease symptoms, this strain caused a significant reduction in pathogen biomass in tomato stems. That could have arisen from the suppressed fungal mycelium by DD6 that clogs the xylem vessels resulting in wilting symptoms in plants (Di Pietro et al., 2003).

Many *Bacillus* strains isolated from various ecological niches have been identified as potential biocontrol agents. However, only a limited number have been reported to be inducers of plant immunity as their main mode of action (Köhl et al., 2019). In this study, we found that pretreatment with EC9 mediate protection against Fusarium wilt of tomato (**Figure 1**). Remarkably, EC9 does not exhibit fungi-toxic activity against *Fol in vitro* and does not possess BGCs, associated with antimicrobial compounds, as predicted using antiSMASH analysis (Madriz-Ordeñana et al., 2022). This suggests that EC9-mediated protection is based on enhanced defense rather than direct antagonistic effects. Furthermore, we did not observe significant growth promoting activity in EC9-treated tomato plants (**Figure 1**). This is in contrast to other *Bacillus cereus* strains, such as AR156 and YN917, that have been shown to promote plant growth in different species (Niu et al., 2011; Zhou et al., 2021).

The phytohormone SA and JA/ET signaling pathways have fundamental roles in the regulation of plant immunity (Glazebrook, 2005; Bari and Jones, 2009; Pieterse et al., 2014). Although the general concept that JA/ET-mediated activation of ISR is triggered by root-associated beneficial bacteria, it has been shown that, depending on host-bacteria-pathogen interactions and experimental conditions, *Bacillus* species can also trigger SA-mediated defense pathways. It is in addition known that these pathways are interconnected in signaling networks, but the relationship between beneficial microorganisms, harmful pathogens and these pathways is not strictly defined (Spoel and Dong, 2008; Thaler et al., 2012). Treatment with *B. cereus* AR156 by soil drenching increased *PR1* transcript level, ROS accumulation, and callose deposition in *A. thaliana*, and it improved the level of defense against the necrotrophic pathogen *Botrytis cinerea* independently of JA/ET and NPR1-mediated signaling (Nie et al., 2017). Additionally, SA-dependent signaling is also required for AR156-mediated protection against the hemi-biotrophic pathogen *Pseudomonas syringae* pv. *tomato* DC3000 (Niu et al., 2011). Extracellular polysaccharides of this bacterium were suggested to confer MAMP-mediated activation of ISR (Jiang et al., 2016). Phthalic acid methyl ester secreted by *B. subtilis* IAGS174 (Akram et al., 2015) and phenylacetic acid secreted by *B. fortis* IAGS162 (Akram et al., 2016) have been proposed as potential ISR activators alleviating the symptoms caused by *Fol*. To determine the involvement of the SA pathway in EC9-mediated protection against *Fol*, we examined the *NahG* transgenic line that is compromised in the accumulation of SA. As shown previously (Di et al., 2017; Constantin et al., 2019), we found that *NahG* makes the plants more susceptible to *Fol* (**Figure 2**). However, EC9-mediated protection was not abolished in *NahG* plants, suggesting that it is SA-independent (**Figures 2A–C**). We also tested the transcript levels of the SA biosynthesis-related *ICS* and *PAL* genes in cv. Moneymaker. The unaffected *ICS* expression and the increased transcript level of *PAL* in *Fol*-inoculated plants at 14 dpi (**Figures 2E, F**) are consistent with the findings of Constantin et al. (2019). The transcript profile for *ICS* agrees with our argument that EC9-mediated defense does not occur through the SA pathway (**Figure 2E**). However, because PAL transcript level is increased by EC9 treatment alone and following pathogen infection at 3 dpi, it can be speculated that SA signaling may still function in an EC9-mediated defense against *Fol* in tomato (**Figure 2F**).

To test the involvement of the phytohormones JA and ET in EC9-mediated protection against *Fol*, we used the *spr2* mutant and the *ACD* OE line, which are deficient in JA biosynthesis and ET production, respectively. We found that pretreatment with EC9 provided protection against the Fusarium wilt of tomato in the corresponding wildtype cultivars. Since protection by EC9 was ineffective in both *spr2* and *ACD* OE lines, we suggest that EC9 activates a defense that is dependent on the JA and ET pathways (**Figures 3A, B**; **Figures 4A, B**). Similar findings have been reported for different *Bacillus* spp. in *Arabidopsis* (Nie et al., 2017) and tomato (Yan et al., 2002). Interestingly, Wang et al. (2018) demonstrated in *Arabidopsis* that *B. cereus* AR156, which is genetically clustered within the *B. cereus sensu lato* group together with EC9 (Madriz-Ordeñana et al., 2022), activates pattern-triggered immunity in a SA, JA, and ET-independent manner. The *PI-I* and *ETR4* genes are fundamental for JA- and ET-dependent signaling, respectively, and they have been reported as marker genes for related pathways in tomato-*Fol* interactions (Di et al., 2017; Constantin et al., 2020). We tested the transcript levels of *PI-I* and *ETR4* in cv. Moneymaker plants, either treated with EC9 or mock, and subsequently challenged with *Fol*. Our results agree with those of Di et al. (2017) who reported that *PI-I* expression was not affected by *Fol* inoculation alone, whereas *ETR4* expression was up-regulated. Noteworthy, EC9 alone did also not induce accumulation of the *PI-I* and *ETR4* transcript prior to *Fol* inoculation. However, the *PI-I* transcript level was augmented following the *Fol* challenge in EC9-treated plants. Similarly, although *ETR4* expression was upregulated in *Fol-*inoculated plants, its expression level was significantly higher when EC9-treatment was combined with *Fol* inoculation (**Figures 3E**, **4E**). While the transcripts are quantified in the stem tissue of plants root-treated with EC9, these results suggest that EC9 systemically primes tomato plants for enhanced expression of *PI-I* and *ETR4*. This is in line with the defense gene priming effects of *B. cereus* AR156 (Niu et al., 2011; Wang et al., 2013; Wang et al., 2014). Madriz-Ordeñana et al., (2022) also proposed that EC9-mediated enhanced defense in Kalanchoe against *Fusarium oxysporum* is associated with defense priming.

Deposition of callose at the site of pathogen attack is one of the early defense responses and has been well documented to play an important role in effective inhibition of pathogen invasion (Luna et al., 2011; Ellinger et al., 2013). MAMP-elicited callose deposition is considered a marker of PTI not only in leaves but also in roots (Luna et al., 2011; Li et al., 2016; Tran et al., 2017). In addition to PAMPs, such as purified flg22 or chitin from pathogenic bacteria or fungi, elicitors like peptidoglycans from root-associated beneficial rhizobacteria *Bacillus subtilis* were shown to induce callose deposition in roots (Millet et al., 2010; Newman et al., 2013). In the present study, we demonstrated that EC9 treatment induces callose deposition in roots. However, we did not find significant differences in the number of callose spots between mock-treated and EC9-treated plants when inoculated with *Fol* (**Figure 5**), suggesting that EC9 treatment does not predispose the host plant for increased callose deposition following *Fol* inoculation. Nevertheless, while the increased callose deposition in EC9-treated roots is an indication of PTI, we speculate that other PTI-related defense responses rather than callose deposition might play a role in the suppression of the *Fol.* We suggest that the systemic priming effect that we observe on the *PI-I* and *ETR4* marker transcripts in stem tissue is linked to a role JA and ET signaling in the protection against *Fol*. Interestingly, colonization of *Arabidopsis* roots by the related strain AR156 does not systemically induce callose deposition in the leaves. Yet, it primes plants for enhanced callose deposition after pathogen attack (Niu et al., 2011; Nie et al., 2017). Furthermore, this strain is capable of inducing callose deposition when infiltrated into *Arabidopsis* leaves (Wang et al., 2018).

## 5 Conclusion

This study provides evidence that the non-antifungal EC9 has strong potential for priming plant immunity against *Fol* in tomato in a JA/ET signaling-dependent manner. These findings provide new insights into the molecular mechanism of EC9-activated protection towards Fusarium. Furthermore, our results indicate that activation of induced resistance through priming of defense mediated by beneficial microorganisms is an interesting approach for identifying new biological control agents with alternative modes of action.

## Data availability statement

The original contributions presented in the study are included in the article/**Supplementary Material**. Further inquiries can be directed to the corresponding author.

## Author contributions

SP, KM-O, and HT-C: conceptualization. SP and KM-O: methodology, investigation and data curation. SP: formal analysis. SP and HT-C: funding acquisition. HT-C and KM-O: supervision. SP: writing-original draft: SP, KM-O, HT-C: writing-review and editing. All authors have read and agreed to the published version of the manuscript.
