# Supplementary material for: Bacillus cereus EC9 protects tomato against Fusarium wilt through JA/ET-activated immunity
Source: Front Plant Sci. 2022 Dec 15;13:1090947. doi: 10.3389/fpls.2022.1090947 (PMC9798288; doi:10.3389/fpls.2022.1090947)
Supplement: Supplementary file 1 [file Table_1.docx]

Supplementary Material

**Supplementary Table 1.** Primer sequences used in this study.

| Gene | Organism | Primer name | Forward primer (5`-3`) | Reverse primer (5`-3`) | Reference |
| --- | --- | --- | --- | --- | --- |
| α-tubulin | Tomato | FP:2149; FP:2150 | TCGTGGCCACTATACCATTG | AGTGACCCAAGACCTGAACC | Constantin et al., 2019 |
| *ETR4* | Tomato | FP:6795; FP:6796 | GGTAATCCCAAATCCAGAAGGTTT | CAATTGATGGCCGCAGTTG |  |
| *PAL* | Tomato | FP:5056; FP:5057 | CGTTATGCTCTCCGAACATC | GAAGTTGCCACCATGTAAGG |  |
| *PI-I* | Tomato | FP:5864; FP:5865 | GTGTACCAACAAAGCTTGCTAAAGA | GTACAACAACACCCAAAATGTTGTC |  |
| *ICS* | Tomato | FP:7620; FP:7621 | TCCAGGCTGAAGATGATGAG | TTATTCCAACCGCAAATTCA |  |
| Plant tubulin | Tomato | FP:2147; FP2148 | CAGTGAAACTGGAGCTGGAA | TATAGTGGCCACGAGCAAAG | Constantin et al., 2020 |
| *SIX8* | *Fol* | FP:6994; FP:7569 | GTATGTCTGATTCTCATGAATA | GTTATGCAGGCGAGTAAAATG |  |
